# A Clinical Assessment of the Outcomes of Intrauterine Insemination Based on Mid-cycle Luteinizing Hormone Levels

**DOI:** 10.7759/cureus.83932

**Published:** 2025-05-11

**Authors:** Hadia Riaz, Fatima Bibi, Erum Gul, Soobia Pathan, Marvi Memon, Shama Chaudhry, Laiba Bhurgari Baloch, Maryum Sana, Ome Kulsoom

**Affiliations:** 1 Obstetrics and Gynaecology, Ziauddin University, Karachi, PAK; 2 Pharmacology and Therapeutics, Liaquat Institute of Medical and Health Sciences, Jamshoro, PAK; 3 Nursing, Akhtar Saeed Medical and Dental College, Lahore, PAK

**Keywords:** fertility, intrauterine insemination, lh surge, luteinizing hormone, ovulation, pregnancy outcomes, reproductive hormones

## Abstract

Background

The reproductive system and the ovulation process depend heavily on the function of the luteinizing hormone (LH). The effect of irregular LH concentrations will impact how follicles mature, affect the release of oocytes, and impact the overall fertility outcomes. This study used clinical data to determine how LH levels affect the effectiveness of intrauterine insemination (IUI) to improve the assessment of fertility treatments.

Methods

Eighty women aged 25-40 years were enrolled in this observational study during their IUI treatment from February to August 2024. Serum testing tracked LH levels from baseline until the middle of the menstrual cycle for all the participants. We tracked essential data points, such as endometrial thickness, follicular dimensions, and the timing for the ovulation trigger. The study evaluated clinical pregnancy outcomes while investigating potential correlations between LH levels and follicle maturity, as well as the effect of LH measurements on improving the IUI timing. The research used t-tests and Pearson's correlation for statistical analysis.

Results

Women who experienced mid-cycle LH peaks within the recommended range achieved a clinical pregnancy rate of 23 (29.1%), which outperformed both low and high LH level groups (p=0.02). The results demonstrated that a timely LH surge positively correlated with mature follicle formation (r=0.36, p=0.008). Women who either failed to produce an LH surge or had early LH surges experienced decreased chances of successful conception.

Conclusion

The success rate of IUI depends heavily on the LH levels of patients. Clinical results for IUI patients can be improved by monitoring and optimizing LH timing for a better fertility response.

## Introduction

Intrauterine insemination (IUI), an assisted reproductive method, is performed routinely as it delivers infertility treatment to couples through an inexpensive, minimally invasive approach [1]. The success of IUI relies significantly on the clinical accuracy of ovulation timing because reproductive hormones manage this process through extensive network interactions [2]. The luteinizing hormone (LH) functions as the regulating hormone which directs ovarian follicle maturation until it starts the ovulation process [3]. The LH surge functions as the principal determinant of the optimal time window for successful fertilization during insemination [4].

If the LH is released at the wrong time, it can reduce the likelihood of pregnancy [5]. Experts are actively researching LH testing as a predictor for the success of IUI, since its clinical value is established [6]. Research on the dynamic patterns of LH levels and their effect on fertility outcomes will help create better strategies to enhance the success rates of IUI [7,8].

In this study, we wanted to assess whether LH hormone measurement, along with its mid-cycle surge, directly impacts IUI outcomes. This observational study evaluates LH as both an indicator for the timing of the IUI procedure and a predictor of clinical success by examining reproductive factors with hormonal assays. Improved hormonal control during fertility treatments in patients can enhance treatment efficacy.

## Materials and methods

We conducted an observational clinical study to evaluate the effects of LH levels on IUI success rates during fertility treatments. The research was conducted on 80 female patients between 25 and 40 years of age from February to August 2024. Subjects were enrolled from gynecology outpatient clinics and fertility centers after obtaining their consent. We included participants who reported more than 12 months of infertility combined with regular menstrual cycles lasting between 25 to 35 days and non-use of hormonal therapy or ovulation-inducing drugs for at least three months. The selected participants also did not suffer from endocrine disorders, autoimmune diseases, or conditions related to reproductive function, and were neither pregnant nor breastfeeding. 

We divided the participants into separate groups based on their mid-cycle LH levels. We defined the optimal LH surge value as ≥17 mIU/mL, and it was measured in the mid-cycle samples from the patients within 24 to 36 hours before administering the ovulation trigger. Serum LH measurements below this limit, which surged before the expected timing, qualified as non-optimal results. We started ovulation when the main follicle reached 18 mm or more, and then performed IUI 24 to 36 hours after giving the trigger shot.

Data gathered from the patients included basic clinical information such as age, BMI, duration of fertility, and its specific cause, along with an assessment of the hormonal profile. The participants also underwent multiple transvaginal ultrasound examinations for the assessment of their ovarian follicles as well as the thickness of the endometrial tissue. Lastly, laboratory testing for estradiol and progesterone occurred during the mid-cycle examination. The pregnancy outcomes were checked by performing serum beta-human chorionic gonadotropin (β-hCG) tests and ultrasound screenings at week six following the embryonic transfer.

All statistical analyses were performed using IBM SPSS Statistics for Windows, Version 27 (Released 2020; IBM Corp., Armonk, New York, United States). Quantitative variables were expressed as mean ± standard deviation (SD). Independent samples t-tests were used to compare continuous variables (e.g., mid-cycle LH levels, endometrial thickness, percentage of mature follicles) between groups with optimal and non-optimal LH surges. To assess the relationship between LH levels and follicular maturity, Pearson’s correlation coefficient was applied. A p-value of <0.05 was considered statistically significant. 

## Results

The study analyzed the effect of LH on IUI results through the assessment of 80 women between 25 to 40 years old from different clinics. The test subjects received both hormonal testing and a detailed assessment of their follicles during their IUI treatment. The average age of the participants was 32.1 (±4.3) years. Table 1 shows the key demographic data and reproductive measurements of the participants, including the follicle size at baseline.

**Table 1:** Baseline demographic and reproductive characteristics of the study population (n=80) Baseline serum LH levels were measured during days 2–3 of the cycle. Descriptive statistics were used. BMI: Body Mass Index; LH: Luteinizing hormone; SD: Standard deviation

Variable	Mean±SD	Range
Age (years)	32.1±4.3	25–40
BMI (kg/m²)	23.9±2.8	19.7–30.1
Baseline LH (mIU/mL)	5.2±1.9	2.1–9.8
Dominant follicle size (mm)	17.4±2.1	14–22

The LH baseline data demonstrated normal clinical values. A typical pattern of ovulation existed in the study participants, as demonstrated by their normal endometrial growth and follicle measurements. Table 2 provides information about LH surge timing and magnitude. We used the baseline values as the starting point for evaluation.

**Table 2:** Characteristics of the luteinizing hormone (LH) surge and the associated outcomes Optimal LH surge ≥17 mIU/mL measured within 24–36 hours before the ovulation trigger. The LH surge values below this threshold were considered non-optimal. Independent t-tests were used. SD: Standard deviation

Parameter	Optimal LH surge group (n=36; Mean±SD)	Non-optimal LH surge group (n=44; Mean±SD)	p-value
Mid-cycle LH (mIU/mL)	18.7±3.5	9.3±4.1	<0.01
Endometrial thickness (mm)	9.2±1.1	8.3±1.3	0.03
Mature follicles (%)	73.5±11.8	61.2±12.4	<0.01

The results from the Pearson's correlation analysis confirmed that patients who experienced stronger mid-cycle LH surges achieved higher rates of mature follicle formation with a correlation coefficient of r=0.36 and a significant statistical value of p=0.008. The LH surge levels and timing in the women matched the 24- to 36-hour interval, leading to statistically significant advances in endometrial growth and follicular development. Table 3 shows a comparison of the values between the pregnant and the non-pregnant groups.

**Table 3:** Luteinizing hormone (LH) levels correlated with pregnancy outcomes Pregnancy was confirmed six weeks after IUI. Serum β-hCG testing and ultrasound were used for detection. Independent t-tests were used. β-hCG: beta-human chorionic gonadotropin

Variable	Pregnant group (n=23)	Non-pregnant group (n=57)	p-value
Mid-cycle LH (mIU/mL)	19.4±3.1	15.2±4.6	0.02
Mature follicles (%)	75.1±9.6	65.7±13.1	0.01
Timing of the ovulation trigger (hrs)	35.3±2.4	38.7±3.1	0.04

The participants who became pregnant had elevated mid-cycle LH levels, and showed improved ovarian follicular maturation. A key factor for IUI success depended on implementing the ovulation triggers at the optimal time, which were derived by monitoring the LH surge. Statistical analysis confirmed that the LH function served as a crucial indicator for successful reproductive outcomes.

## Discussion

This study examined how mid-cycle LH levels influenced IUI outcomes in women. Our study showed that properly timed LH release increases the development of follicles and produces ideal endometrial growth conditions, leading to better pregnancy outcomes. It also demonstrated that mid-cycle LH measurement provides an accurate prognostic value for IUI success, as it indicates the optimal timing for initiation of ovulation. An optimal physiological LH surge produced significantly more mature follicles, coupled with thickened endometrial lining that represented established reproductive health markers [9,10]. These observations correspond with previous studies, which demonstrated that LH was the regulatory hormone coordinating follicular development along with endometrial maturation [11].

The central role of LH in ovulation becomes apparent through its positive relationship with mature follicle development and demonstrates its clinical value as a fertility indicator [12]. Pregnancy success rate reached 29.1% among participants who experienced on-time LH surges, and aligns with published research on how the timing of the LH surge improves conception possibilities [13]. Research findings confirmed how inadequate or non-existent LH surges decrease the accuracy of the IUI timing, which results in less mature oocytes and diminished implantation prospects [14]. Research revealed that endometrial thickness stood as a critical factor for predicting pregnancy outcomes to supplement the data from hormonal and follicular analysis. A receptive endometrial environment requires hormonal cues from LH and estrogen to function properly for embryo implantation [15]. The findings of the study proved that the success of assisted reproduction depends on the collaborative influence between endocrine factors and morphological parameters [16].

The results of this observational study supported other experimental research showing that IUI triggered by an LH surge produces better clinical outcomes than timing based only on observations or hCG administration. To achieve the best implantation and pregnancy outcomes, scientific evidence indicates that LH surge detection must coincide with follicular development and endometrial maturity [17]. Current research shows that inadequate LH monitoring results in missed fertilization time. The combination of the morphological and hormonal assessment encourages the creation of novel IUI treatment strategies [18]. The current understanding of hormones and fertility care strengthens the case for using exact hormonal monitoring in reproductive treatment.

We obtained useful data from this study, but also acknowledge several limitations. The overall validity of results was limited by the small sample size and the brief observation period. The data on hormonal dynamics was collected at only two points during the cycle, thereby interrupting the measurement of the fluctuations during the full cycle. Future studies should perform regular hormone testing over time to assess whether the LH trigger threshold should be adjusted for different patient groups to improve the timing and outcomes. 

## Conclusions

This study establishes that mid-cycle LH levels are a key predictor for the success of IUI. Optimal LH surges were significantly associated with mature follicle development, increased endometrial thickness, and higher pregnancy rates. The precise timing of insemination with distinctive hormonal signals increases the likelihood of success for patients with unexplained infertility or irregular cycles. The use of personalized protocols based on LH levels has the potential to significantly improve reproductive results. 

Subsequent research should target refinement of the LH-defined boundaries alongside continuous hormonal tracking and further customization of the ‘ovulation’ triggers. Incorporating these methods into standard clinical practice may facilitate the enhancement of clinical and patient satisfaction in assisted reproductive techniques.

## References

[1] Nesbit CB, Blanchette-Porter M, Esfandiari N (2022). Ovulation induction and intrauterine insemination in women of advanced reproductive age: a systematic review of the literature. J Assist Reprod Genet.

[2] Kuru Pekcan M, Tokmak A, Ulubasoglu H, Kement M, Özakşit G (2023). The importance of infertility duration and follicle size according to pregnancy success in women undergoing ovulation induction with gonadotropins and intrauterine insemination. J Obstet Gynaecol.

[3] Oduwole OO, Huhtaniemi IT, Misrahi M (2021). The roles of luteinizing hormone, follicle-stimulating hormone and testosterone in spermatogenesis and folliculogenesis revisited. Int J Mol Sci.

[4] Erden M, Mumusoglu S, Polat M, Yarali Ozbek I, Esteves SC, Humaidan P, Yarali H (2022). The LH surge and ovulation re-visited: a systematic review and meta-analysis and implications for true natural cycle frozen thawed embryo transfer. Hum Reprod Update.

[5] Pimolsri C, Lyu X, Goldstein C (2021). Objective sleep duration and timing predicts completion of in vitro fertilization cycle. J Assist Reprod Genet.

[6] Zippl AL, Wachter A, Rockenschaub P, Toth B, Seeber B (2022). Predicting success of intrauterine insemination using a clinically based scoring system. Arch Gynecol Obstet.

[7] Esencan E, Beroukhim G, Seifer DB (2022). Age-related changes in folliculogenesis and potential modifiers to improve fertility outcomes - a narrative review. Reprod Biol Endocrinol.

[8] Cunha TO, Martins JP (2022). Graduate student literature review: effects of human chorionic gonadotropin on follicular and luteal dynamics and fertility in cattle. J Dairy Sci.

[9] Chen L, Jiang S, Xi Q, Li W, Lyu Q, Kuang Y (2023). Optimal lead follicle size in letrozole human menopausal gonadotrophin intrauterine insemination cycles with and without spontaneous LH surge. Reprod Biomed Online.

[10] Esmailzadeh S, Faramarzi M (2007). Endometrial thickness and pregnancy outcome after intrauterine insemination. Fertil Steril.

[11] Han S, Liu MH, Lv YS, Ren HY, Guo J, Li Y, Liu S (2022). Effects of low luteinizing hormone during ovarian stimulation on endometrial gene expression and function - transcriptome analysis during the implantation window. Reprod Sci.

[12] Huniadi A, Bimbo-Szuhai E, Botea M (2023). Fertility predictors in intrauterine insemination (IUI). J Pers Med.

[13] Kucuk T (2008). Intrauterine insemination: is the timing correct?. J Assist Reprod Genet.

[14] Potapragada NR, Babayev E, Strom D, Beestrum M, Schauer JM, Jungheim ES (2023). Intrauterine insemination after human chorionic gonadotropin trigger or luteinizing hormone surge: a meta-analysis. Obstet Gynecol.

[15] Bai X, Zheng L, Li D, Xu Y (2021). Research progress of endometrial receptivity in patients with polycystic ovary syndrome: a systematic review. Reprod Biol Endocrinol.

[16] Huang X, Sun Q, Tang X (2023). Factors influencing the pregnancy outcome of intrauterine insemination and follow-up treatment. J Hum Reprod Sci.

[17] Jiang S, Chen L, Gao Y, Xi Q, Li W, Zhao X, Kuang Y (2022). The effect of spontaneous LH surges on pregnancy outcomes in patients undergoing letrozole-HMG IUI: a retrospective analysis of 6,285 cycles. Front Endocrinol (Lausanne).

[18] Maman E, Adashi EY, Baum M, Hourvitz A (2023). Prediction of ovulation: new insight into an old challenge. Sci Rep.

